# Cationic Albumin Encapsulated DNA Origami for Enhanced Cellular Transfection and Stability

**DOI:** 10.3390/ma12060949

**Published:** 2019-03-21

**Authors:** Xuemei Xu, Shiqi Fang, Yuan Zhuang, Shanshan Wu, Qingling Pan, Longjie Li, Xiaohui Wang, Xueqing Sun, Bifeng Liu, Yuzhou Wu

**Affiliations:** 1 Britton Chance Center for Biomedical Photonics at Wuhan National Laboratory for Optoelectronics, Hubei Key Laboratory of Bioinorganic Chemistry and Materia Medica, School of Chemistry and Chemical Engineering, Huazhong University of Science and Technology, Wuhan 430074, China; xuxuemei@hust.edu.cn (X.X.); fangshiqi@hust.edu.cn (S.F.); zhuangyuan@hust.edu.cn (Y.Z.); wushanshan@hust.edu.cn (S.W.); panqingling@hust.edu.cn (Q.P.); lilongjie18@hust.edu.cn (L.L.); m201670275@hust.edu.cn (X.W.); sunxueqing@hust.edu.cn (X.S.); bfliu@hust.edu.cn (B.L.); 2 Max Planck Institute for Polymer Research, Ackermannweg 10, 55128 Mainz, Germany

**Keywords:** cationic albumin, DNA origami, cellular uptake, stability

## Abstract

DNA nanostructures, owing to their controllable and adaptable nature, have been considered as highly attractive nanoplatforms for biomedical applications in recent years. However, their use in the biological environment has been restricted by low cellular transfection efficiency in mammalian cells, weak stability under physiological conditions, and endonuclease degradation. Herein, we demonstrate an effective approach to facilitate fast transfection of DNA nanostructures and enhance their stability by encapsulating DNA origami with a biocompatible cationic protein (cHSA) via electrostatic interaction. The coated DNA origami is found to be stable under physiological conditions. Moreover, the cHSA coating could significantly improve the cellular transfection efficiency of DNA origami, which is essential for biological applications.

## 1. Introduction

The DNA origami technique, which creates highly defined-complex nanostructures through programmable folding of a long circular single strand DNA molecule, has been experiencing remarkably fast developments in the last ten years [1,2,3]. DNA origami nanostructures have the ideal size (10 to 100 nm) for nanomedicine applications. They are biodegradable and biocompatible, and their application in diagnostics and therapeutics has been highlighted [4,5,6]. In comparison to conventional materials used for nanomedicine, such as polymeric or inorganic nanoparticles, DNA nanostructures benefit from highly controlled size and shape, precise modifications, programmable responsiveness, and the possibility of designing sophisticated transformations for smart drug delivery [7,8,9,10]. Many studies have already demonstrated these advantages. For instance, the triangular DNA nanostructure has been shown as a biocompatible drug carrier for tumor targeting [7]. DNA nanorobot could transform and release cargos in response to several bio-signals and even allow logical calculations [8,9]. Moreover, DNA tubes opening up specifically in tumors could induce intravascular thrombosis in tumor-associated blood vessels in vivo [10].

However, the potential for biomedical applications of DNA nanotechnology has been limited due to the instability of DNA nanostructures and their low efficiency for entering mammalian cells. Stabilization of most DNA nanostructures typically requires divalent cations (e.g., Mg^2+^) to overcome electrostatic repulsion between closely packed phosphate anions on DNA backbone [11,12,13,14]. Therefore, depending on the DNA origami design, most DNA nanostructures are highly sensitive to the buffer environment. Particularly the low cation concentrations and various pH levels in physiological fluids often challenges DNA origami stability. Moreover, the nuclease in vitro and in vivo would result in rapid degradation of DNA nanostructures, which also hinders the desired function of DNA nanorobots [15,16]. In addition, since the mammalian cell membrane often exhibits negative charges, the charge repulsion restricts the transfection efficiency of DNA nanostructures. Therefore, to allow DNA nanostructures to properly function as intelligent nanorobots in living organisms, facile methods to improve their stability and cell transfection efficiency are in high demand.

Several approaches have been investigated to address these challenges, including encapsulating the DNA nanostructure into liposome [17], coating them with cationic or amphiphilic polymers [18,19,20,21,22], and chemical modifications of DNA backbone [23]. However, these studies were inevitably introduced non-biodegradable synthetic molecules, which compromise the advantages of DNA nanostructures as a biodegradable and biocompatible nanoplatform. While studies have shown that cationic proteins such as virus capsids are biocompatible tools for coating DNA origami, which resulted in an enhanced cell transfection [24], their sophisticated preparation and in vivo immunogenicity could not be simply overlooked. Also, a synthetic cationic protein for DNA origami coating was prepared by conjugating cationic dendron to protein [25]. However, the dendron molecule again introduced a non-biodegradable component in this system. Recently, albumin protein, the most abundant serum protein, has been investigated for DNA origami protection [26]. To render effective interaction between these two both negatively charged entities, albumin protein has to be conjugated with a cationic dendron molecule or attached to DNA structure via a hydrophobic alkyl chain modified DNA anchor. Similarly, these synthetic moieties also introduced non-biodegradable components. Furthermore, the sophisticated preparation of such complex dendron-protein conjugates or alkyl chain modified DNA structures also limits their broad applications. 

**Figure 1:**
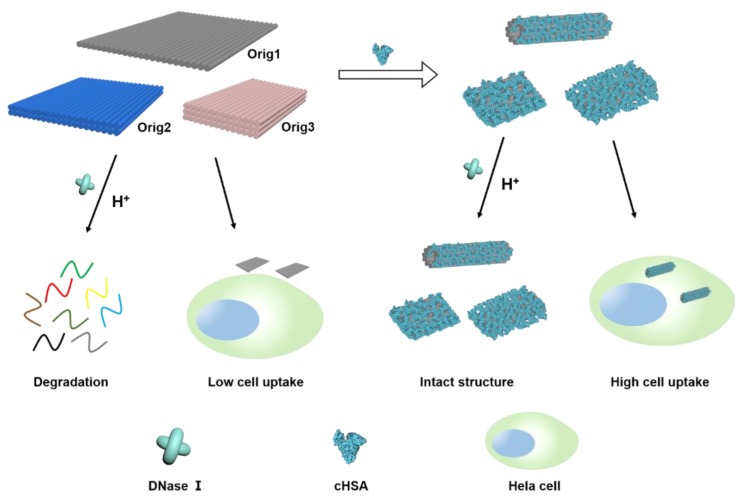
Schematic of cHSA coating strategy for enhancing the stability and transfection efficiency of DNA origami. DNA origami was coated by cHSA via electrostatic interaction between the negatively charged DNA origami and positively charged cHSA. Plain DNA origami was degraded by DNase I and dissociated at acidic pH, whereas coated origami was found to be resistant to DNase I digestion and stable under the acidic condition. The coated DNA origami nanostructures could also be transfected to cells more efficiently.

Taking into account the considerations of facile preparation, economic practicality and biodegradability, herein, we disclose our studies using cationic human serum albumin (cHSA) as an easily accessible cationic protein for coating DNA origami to improve their stability and transfection efficiency. cHSA was obtained by one step modification of human serum albumin, the most abundant protein in the blood-stream that widely used for biomedical applications [27,28]. Many researchers, including us, have shown that modified albumin derivatives, such as cHSA and cHSA-conjugates, have good biocompatibility and biodegradability, and they could serve as promising carriers for gene delivery and drug delivery [29,30,31,32]. Due the fact that the positive charges of cHSA are diluted by other neutral and negatively charged groups on the surface, they exhibit the lowest toxicity and most promising biocompatibility compared to other cationic macromolecules such as polyethylenimine, poly(L-lysine). The coating of cHSA onto DNA origami can be instantly accomplished by simply mixing the two components, and this process is driven by electrostatic interaction. This coating can protect the DNA origami structures from endonuclease digestion, and even allow DNA origami to be stable in low salt concentration and acidic condition. Besides, the cationic coating also facilitates efficient transfection of DNA origami into mammalian cells (Figure 1). In comparison to the reported methods for DNA origami coating, our strategy using cHSA is easier, scalable, and biocompatible, which is anticipated to broaden applications of DNA origami in the biomedical area.

## 2. Materials and Methods

### 2.1. Materials

All reagents are commercially available and used without any further purification. P8064 DNA scaffold DNA was purchased from tilibit nanosystems^®^ GmbH (Garching, Germany). DNA staple strands were purchased from Wuhan GeneCreate Biological Engineering Co., Ltd. (Wuhan, China). Tris(hydroxymethyl)aminomethane and Ethylenediaminetetraacetic acid disodium salt were purchased from BEIJING LIUYI BIOTECHNOLOGY CO., LTD (Beijing, China). Acetic acid and Magnesium chloride hexahydrate (MgCl_2_·6H_2_O) were purchased from Sinopharm Chemical Reagent Co., Ltd. (Shanghai, China). Agarose and 5 × TBE for electrophoresis were purchased from Sangon Biotech Co., LTD (Shanghai, China). GelRed was purchased from biosharp. 1-Ethy-3-(3-dimethylaminopropyl) carbodiimide hydrochloride (EDC·HCl) and Human Serum Albumin (HSA) were purchased from Sigma-Aldrich. Hoechst33342 and Cell Mask Green were purchased from Thermo Fisher Scientific. Carbon grid was purchased from Beijing XXBR Technology Co., Ltd. (Beijing, China). Uranyl acetate solution was purchased from Beijing Zhongjingkeyi Technology Co., Ltd. (Beijing, China). Distilled water (18.2 MΩ·cm, MilliQ system) was used for all experiments.

### 2.2. Fabrication of DNA Origami with Different Structures

Single-layer DNA origami (**Orig1**) folding and purification: The **Orig1** was assembled by mixing M13mp18 phage scaffold DNA with desired staple strands (1:10) in 1×TAE/Mg buffer (20 mM Tris, 1 mM EDTA, 10 mM Acetic acid, 12 mM Mg^2+^, pH 8.0) and annealing from 70 °C to 20 °C over 1.5 h. The excess staples were removed by the PEG precipitation method. Briefly, the reaction solution containing DNA origami and excess staples was mixed with the same volume of PEG solution (15% PEG8000, 5 mM Tris, 1 mM EDTA, 505 mM NaCl) and centrifuged at 12,000× *g*, 23 °C for 30 min. The supernatant was removed and the pellet was dissolved in 1×TAE/Mg buffer again.

Multilayer DNA origami folding and purification: The **Orig2** and **Orig3** DNA origami were assembled by mixing P8064 scaffold DNA with desired staple strands (1: 20) in 1×TE/Mg buffer (5 mM Tris, 1 mM EDTA, 16 mM Mg^2+^, pH 8.0) and annealing from 80 °C to 24 °C for 72 h.

### 2.3. Preparation of cHSA and cHSA Coated Origami with Different Ratios

cHSA was prepared according to a previously reported method. Briefly, HSA was dissolved in ethylenediamine-HCl solution (2.5 M, pH 4.75), then EDC was added and the mixture solution was stirred for 75 min. The reaction was then terminated by adding acetate buffer (4 M, pH 4.75). After the reaction, cHSA was washed twice with acetate buffer (4 M, pH 4.75) and three times with deionized distilled water using Vivaspin 20 (30 kDa MWCO) centrifugal concentrator and then lyophilized to obtain cHSA as a white fluffy solid.

To prepare the cHSA coated origami, the DNA origami structures were generally diluted to 0.6 nM in 1×TAE/Mg buffer, followed by the addition of cHSA with increasing amounts to obtain a series of mixtures with different cHSA/origami ratios (50:1, 200:1, 500:1, 1000:1, 2000:1, and 4000:1). Each mixed sample was incubated for 5 min at room temperature to allow the coating of DNA origami by cHSA before analysis. The coating for **Orig2** and **Orig3** was conducted by following the same procedure used for **Orig1**.

### 2.4. Agarose Gel Electrophoresis

Each sample (plain DNA origami, coated origami, coated origami treated with DNase I) was mixed with loading buffer and respectively loaded on 1% agarose gel, and electrophoresis was performed in 0.5×TBE/Mg^2+^ buffer at 90 V for 40 min at room temperature. After running, the gel was stained by GelRed for 40 min before taking images.

### 2.5. Stability of Coated Origami in Low Salt Solution

To test the stability of control DNA origami and coated origami in low salt buffer, the coating (4000:1, 1000:1) process was accomplished in 1×TAE/Mg (12 mM Mg^2+^, pH 8.0) buffer, followed by adding 5 volume of 1×TAE (pH 8.0) buffer to make the final concentration of Mg^2+^ at 2 mM. Then, the final coated origami solution (2 mM Mg^2+^) was incubated at room temperature for 16 h before being loaded on a carbon grid.

### 2.6. Stability of Coated Origami in Weak Acid

To test the stability of control DNA origami and coated origami in weak acid, the coating (4000:1, 1000:1) process was accomplished in 1×TAE/Mg (12 mM Mg^2+^, pH 8.0) buffer, followed by adding 5 volume of 1×TAE/Mg (12 mM Mg^2+^, pH 2.60) buffer to make the final pH as 3.59. Then, the final coated origami solution (weak acid) was incubated at room temperature for 16 h before analysis.

### 2.7. AFM Imaging

The Atomic Force Microscope (AFM) imaging was performed with a Bruker AFM equipped with the ScanAsyst mode in liquid. After clear mica was observed, plain **Orig1** or coated **Orig1** with different ratios were respectively introduced into the liquid pool. After 5 min deposition, the samples were scanned with the scan rates between 1 and 3 Hz. Different images were acquired at different areas of the mica surface to ensure the reproducibility of the results. All images were analyzed by using the NanoScope Analysis 1.50.

### 2.8. TEM Imaging

Sample preparation: increasing amounts of cHSA were respectively added into a constant amount of DNA origami to make the ratio of cHSA/DNA origami to be 50:1, 200:1, 500:1, 1000:1, 2000:1, and 4000: 1. Each mixture was incubated at room temperature for 5 min before loading on the carbon grid. Sample staining: 5 μL of sample solution was placed onto the plasma cleaned (30 s oxygen plasma flash) 300 mesh copper grid for 2.5 min for sample deposition. Then, the excess liquid was drained by filter paper, and the grid was washed with buffer for 10 s. Then, 2.5 μL (3%) of uranyl acetate solution was placed onto the grid for 40 s; the excess solution was removed by filter paper. The samples were imaged after drying. The Transmission Electron Microscope (TEM) images were collected using HITACHI HT7700 operated at an acceleration voltage of 120 kV and analyzed using ImageJ software.

### 2.9. Cell Culture and Confocal Imaging

The Hela cell line was obtained from Shanghai Zhong Qiao Xin Zhou Biotechnology Co., Ltd. (Shanghai, China). Hela cells were cultured in Dulbecco’s modified Eagle’s medium (DMEM) with 10% fetal bovine serum (FBS) and 1% penicillin streptomycin in a culture flask at 37 °C under 5% CO_2_ atmosphere. Hela cells (8000) were seeded on an 8-well confocal chamber and attached for 48 h. Then 200 μL of 0.1 nM **Orig1** and coated **Orig1** (1000:1, 2000:1) samples were, respectively, added into the chamber and incubated with Hela cells for 6 h. The cell nuclei were stained with 0.75 μg/mL Hoechst33342 for 20 min, and cell membrane was stained with 0.5× cell mask green for 10 min at 37 °C before measurement. Laser Scanning Confocal Microscopy (LSCM) imaging: The confocal images were taken with a FV 1000 Olympus biological confocal scanning microscope. 

## 3. Results and Discussion

### 3.1. Coating Behavior of Origami and cHSA

**Figure 2:**
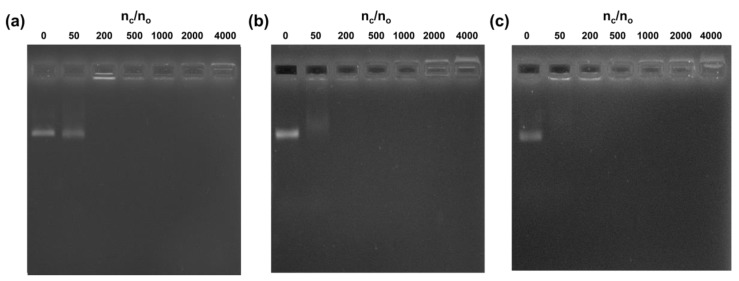

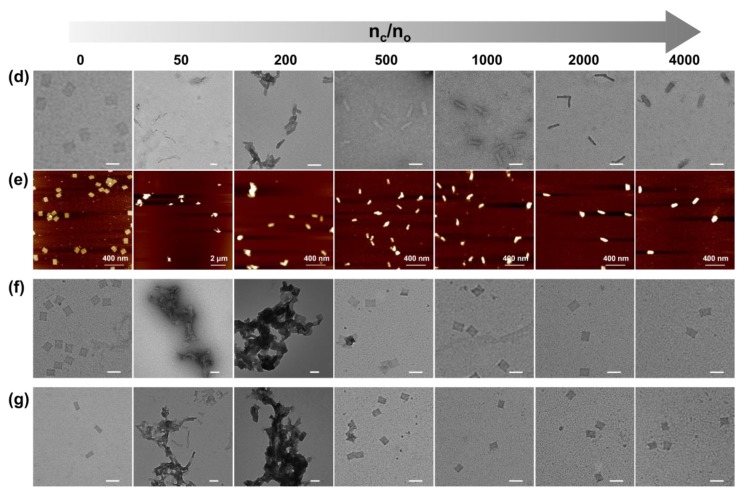
Gel EMSA and TEM, AFM micrographs of the samples. (**a**) Agarose gel EMSA of **Orig1** and coated **Orig1** with increasing ratios of n_c_/n_o_; (**b**) Gel EMSA of **Orig2** and coated **Orig2** with increasing ratios; (**c**) Gel EMSA of **Orig3** and coated **Orig3** with increasing ratios; (**d**) TEM images of **Orig1** and coated **Orig1** with increasing ratios of nc/no; (**e**) AFM images of **Orig1** and coated **Orig1** with increasing ratios; (**f**) TEM images of **Orig2** and coated **Orig2** with increasing ratios of nc/no; (**g**) TEM images of **Orig3** and coated **Orig3** with increasing ratios of nc/no, scale bar in all TEM images is 100 nm.

Three DNA origami nanostructures with different rigidity (shown in Figure S1) were prepared and purified using reported protocols [33,34,35] and analyzed by gel electrophoresis and transmission electron microscopy (TEM) (Figure S2). The **Orig1** was found to be a single layer DNA tile flexible for folding, while the **Orig2** and **Orig3** formed double layers and triple layers structures that are more rigid. The cHSA was prepared by converting the carboxyl groups of amino-acid residue to amino groups via ethylenediamine modification according to literature methods [31]. The molecular weight of cHSA increased from 67 kDa (native HSA) to 71 kDa according to MALDI-Tof spectroscopy (Figure S3a), indicating ~100 of carboxyl groups were converted to amino groups on HSA. The zeta potential of cHSA was measured to be 28.2 mV at pH 7.0 (Figure S3b), which allowed the cHSA to bind and assemble with DNA origami via electrostatic interaction. We first demonstrated that the coating process happens very fast; the complete coating can be observed even in 5 min by real-time imaging through atomic force microscopy (AFM) (Figure S4). Next, cHSA/origami complex was prepared with cHSA to origami ratio (defined as nc/no) as 0, 50, 200, 500, 1000, 2000, and 4000, and each was analyzed by gel electrophoresis, TEM and AFM (**Orig1**) (Figure 2a,d,e). According to gel electrophoresis, the free DNA origami band disappeared when the nc/no ratio increased. Moreover, a new band appeared near the loading well, which can be stained by both Gelred DNA stain and Coomassie blue protein stain (Figure S5). Thus, it should correspond to the cHSA coated DNA origami. The slow mobility of cHSA coated DNA origami is likely ascribed to the neutralization of net charges when mixing the positively and negatively charged species. By further increasing the nc/no ratio, the band corresponding to cHSA/origami complex could even move toward the cathode due to the high amount of positive charges. Noteworthy, when pure cHSA was loaded on agarose gel under the same condition, they were also observed at the loading well slightly moving to the cathode (Figure S6) which is similar with cHSA coated DNA origami at high cHSA ratio. This again supported that the behavior of cHSA/origami complex on agarose gel is due to the property of cHSA, but not aggregation. In the case of **Orig1**, the complete coating (indicated by disappearance of free DNA origami band) can be realized when nc/no ratio is more than 200. This ratio can be reduced to 50 for **Orig2** and **Orig3** due to the decreased surface area (Figure 2b,c,f,g). Noteworthy, although the complete coating of DNA origami can be reached at relatively low nc/no ratio, large aggregations were found on TEM and AFM images in these cases (e.g., nc/no = 200 for **Orig1**, nc/no = 50 and 200 for **Orig2** and **Orig3**). This is due to the insufficient amounts of cHSA that have to be shared between several DNA origami to compensate for the negative charges (Figure S7). By further increasing the nc/no ratio up to 1000, the aggregation was successfully avoided (Figures S8–S10), indicating more cHSA were required to ensure complete passivation of DNA origami surface in order to reach sufficient stability and dispersity. Dynamic light scattering measurements (Figure S11) also supported that, when the cHSA ratio is larger than 200, aggregations could be avoided and the particle size are around 100 nm with good monodispersity. Moreover, according to AFM, the average height of cHSA coated **Orig1** increased with increasing of nc/no from 200 to 2000. However, no difference was observed when the ratio was set at 2000 or 4000; the average height was 15 ± 2 nm in both cases (Figures S12 and S13), suggesting that the coating reached saturation when the ratio is above 2000. Similar observations were also recorded for the length and width of coated **Orig2** and **Orig3** according to TEM, and saturation coating also occurred when the ratio is above 2000 (Figure S14). These results showed that it is necessary to apply cHSA with nc/no between 1000 to 2000 for reliable coating of DNA origami. Moreover, due to the strong electrostatic interaction and the softness of the structure of **Orig1** [36], cHSA coating induced self-folding of **Orig1** into a tube shape (Figure 2d,e). This phenomenon has also been reported when the same DNA structure was interacting with positively charged virus capsid [24]. In contrast, no folding was observed for more rigid **Orig2** and **Orig3** (Figure 2f,g). Furthermore, the coated DNA origami was found to be stable for at least two days according to the TEM (Figure S15). Collectively, these results suggested that cHSA could form a stable coating on DNA origami structures and good solution dispersity could be achieved when sufficient amounts of cHSA are applied.

To probe whether naturally occurring cationic proteins could also coat on DNA origami, we also examined the interaction between DNA origami and avidin (a native cationic protein with isoelectric point = 10). It was found that, the size and shape of DNA origami remained unchanged on TEM (Figure S16a) even after mixing with large amounts of avidin, and the DNA origami band did not shift on agarose gel (Figure S16b). The native albumin was also tested for **Orig1** coating, and no interaction was observed on agarose gel (Figure S17). These results suggested that the highly cationic nature of cHSA (zeta potential = 28.2 mV at pH = 7) is particularly essential for the successful coating of DNA origami, and the coating is likely to be stable even in bloodstream with a high concentration of proteins, since the native proteins are unlikely to be highly positively charged under physiological conditions.

### 3.2. Stability Analysis of Coated Origamis

**Figure 3:**
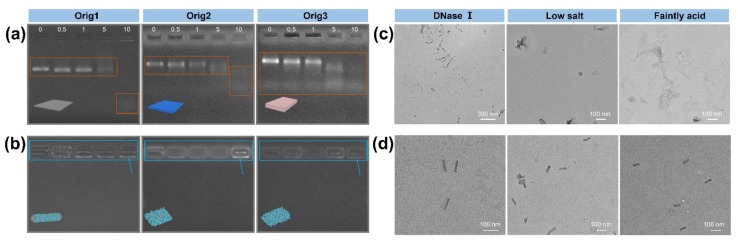
Gel EMSA and TEM test of the stability of DNA origami with and without coating. Gel EMSA of (**a**) plain DNA origami and (**b**) coated origami with nc/no ratio of 1000 treated with DNase I (0, 0.5, 1, 5, 10 Unit in 15 μL of sample volume). TEM images of (**c**) plain **Orig1** and (**d**) coated **Orig1** with nc/no ratio of 4000 after DNase I, low salt concentration, and acid incubation treatment. DNA fragments were highlighted in red pane, cHSA coated origamis-DNase I complexes were highlighted in blue pane.

Since nuclease is widely present in living organisms [11], the degradation of DNA origami by nuclease seriously limits the application of DNA nanostructures in biology. Therefore, the stability of cHSA coated DNA origami against nuclease degradation was analyzed by gel electrophoresis and TEM. A constant amount of DNA origami (**Orig1**, **Orig2**, and **Orig3**) with and without cHSA coating were respectively treated with increasing amount of DNase I at 37 °C for 80 min. As shown in Figure 3a, the appearance of low molecular weight DNA bands and disappearance of origami band indicated clear degradation of uncoated DNA origami when DNase I was up to 5 and 10 Unit. For cHSA coated origami (nc/no = 1000), no low molecular weight DNA bands were observed even with 10 Unit of DNase I and the cHSA/DNA origami complex band remained unchanged. For direct visualization, cHSA coated DNA origami after incubation with 10 Unit of DNase I for 80 min was imaged by TEM, and intact structures were observed for all tested origamis (**Orig1**, **Orig2**, and **Orig3**, Figure S18). The aggregation observed was likely due to the electrostatic interaction between a high concentration of negatively charged DNase I (pI ~ 4.7) and the positively charged cHSA shell. In contrast, the partially coated DNA origamis with nc/no ratio of 50 and 200 were found unstable when treated with DNase I. The free origami band and degradation fragments were all observed (Figure S19a,b). Therefore, it is clear that cHSA coating could efficiently protect DNA origami structures against nuclease degradation.

Except for DNase I degradation, it has been reported that stabilization of most DNA origami structures requires divalent cations (Mg^2+^) to overcome electrostatic repulsion between closely packed negatively charged DNA phosphate anions [12]. Therefore, most DNA origami nanostructures exhibit poor structural integrity in low salt buffers. The application of DNA nanostructures is strongly limited due to the lack of high concentration of Mg^2+^ under physiological condition [37]. Moreover, DNA origami structures have also been shown to be unstable in an acid environment since there are a large number of phosphate anions on DNA backbones. Therefore, the stability of cHSA coated DNA origami (**Orig1** with nc/no ratio of 4000) in a low salt buffer (final concentration of 2 mM Mg^2+^) and a weakly acid buffer (pH 3.59) for 16 hrs was tested. As shown in Figure 3c, the uncoated **Orig1** was not stable under these conditions, and no intact structures could be observed on TEM imaging, starkly contrasting to the coated **Orig1,** maintaining the structural integrity (Figure 3d, Figure S20). Besides, coated **Orig1** with nc/no ratio of 1000 was found not to be stable under the tested low salt condition (Figure S21a), but it was still stable under weak acid conditions (Figure S21b). These results demonstrated that sufficient surface passivation is necessary to obtain high stability of the cHSA coated DNA origami under low salt and acidic conditions.

### 3.3. Cell Uptake of Coated Origami

**Figure 4:**
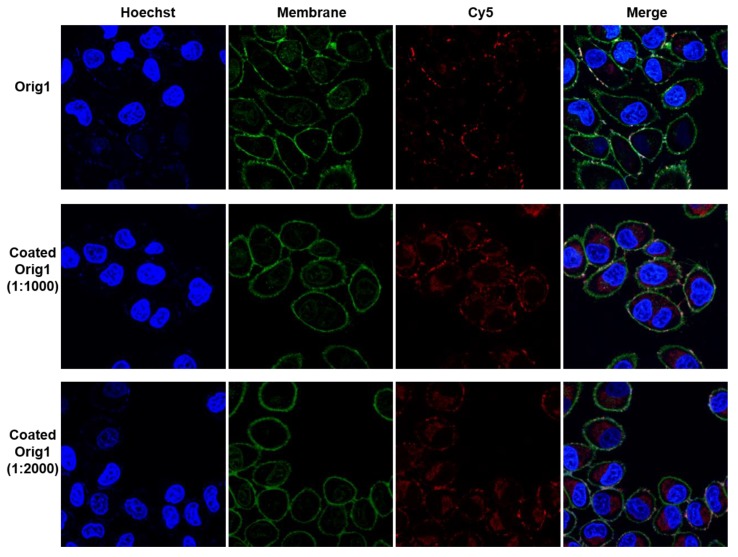
Confocal images of Hela cells incubated with plain **Orig1** and coated **Orig1** with ratio of 1000 and 2000.

Since the membrane of mammalian cells is often negatively charged, DNA nanostructure normally have low transfection efficiency due to electrostatic repulsion. The positively charged cHSA coating is thus expected to enhance the transfection efficiency of DNA origami. To demonstrate this, we first verified that the coating process was achieved in cell culture medium via TEM imaging. The DNA origami **Orig1** was pre-diluted in DMEM cell culture, followed by the addition of increasing amounts of cHSA to obtain the coated origami with nc/no ratio of 4000, 2000, 1000 and 500. The TEM imaging demonstrated that the coating was also effective in low salt physiological condition. Notably, the coated origami was fully aggregated with the ratio of 500, partly aggregated with the ratio of 1000, and fully dispersed with the ratio of 2000 and 4000 (Figure S22), which is consistent with the results in buffer solutions. Based on this result, the transfection efficiency was then examined in vitro. The **Orig1** was labeled with eleven Cy5 (Table S1), and then plain **Orig1** and the coated **Orig1** with nc/no ratio of 1000 and 2000 were respectively incubated with Hela cells for 6 h before imaged by confocal microscopy. As shown in Figure 4, no noticeable cell uptake was observed with uncoated **Orig1**, and **Orig1** obviously aggregated on cell membrane, whereas both cHSA coated **Orig1** showed significantly enhanced cellular uptake. The fluorescence signals were homogeneously distributed in cells, and less aggregation on the cell membrane was observed. We have quantified the red fluorescence inside cells that are not co-localized with membrane staining using Image J (≈18 cells were observed by confocal). Three times enhancement in fluorescence intensity was obtained, supporting the significant increase of cell uptake efficiency with cHSA coating (Figure S23). In addition, no cytotoxicity was found under these conditions (Figure S24). The above results proved that cHSA coating could be an efficient method to improve cell transfection efficiency of DNA origami.

## 4. Conclusions

In summary, we have demonstrated a simple strategy for improving the stability and transfection efficiency of DNA nanostructures. Encapsulating DNA origami with cHSA could be rapidly accomplished in 5 min. When a sufficient amount of cHSA was applied, cHSA/origami complexes exhibited very good dispersity both in buffer and in cell culture medium. The protein shell can completely protect DNA origami from DNase I degradation and enhance the structural integrity under low salt concentration, even in weak acid conditions. The transfection into mammalian cells could also be significantly increased. Therefore, cHSA coatings offer a facile protecting strategy for DNA origami in biomedical applications such as drug delivery, which is fast, effective, and biocompatible.

## Supplementary Materials

The following are available online at https://www.mdpi.com/1996-1944/12/6/949/s1, Figure S1: The folding of **Orig1**, **Orig2** and **Orig3**. Figure S2: Gel and TEM characterization of purified DNA origami structures. Figure S3: MALDI-Tof spectrum and zeta potential of cHSA. Figure S4: AFM real time observation of coated **Orig1**. Figure S5: The cHSA shell was stained with coomassie brilliant blue in agarose gel. Figure S6: Gel EMSA of pure cHSA. Figure S7: Schematic illustration for coating process of different amount of cHSA and DNA origami. Figure S8: TEM images of coated **Orig1** with different ratios. Figure S9: TEM images of coated **Orig2** with different ratios. Figure S10: TEM images of coated **Orig3** with different ratios. Figure S11: DLS measurement of coated **Orig1**. Figure S12: AFM images of coated **Orig1** with different ratios. Figure S13: The measured height of coated **Orig1** with different ratios. Figure S14: The measured length and width of coated **Orig2** and **Orig3**. Figure S15: TEM imaging of the coated **Orig1** in buffer for two days. Figure S16: TEM images and Gel EMSA of complexes of avidin and **Orig1**. Figure S17: Gel EMSA of HSA and **Orig1** complexes. Figure S18: TEM images of coated **Orig2** and **Orig3** treated with DNase I. Figure S19: Gel EMSA of coated **Orig1** with low ratios treated with DNase I. Figure S20: TEM images of coated **Orig1** with ratio 4000 under DNase I treatment, low salt, weak acid conditions. Figure S21: TEM images of coated **Orig1** with ratio 1000 under low salt, weak acid conditions. Figure S22: TEM images of coated **Orig1** with different ratios in cell medium. Figure S23: Relative red fluorescence data in cells of plain origami and coated origami samples. Figure S24: Cytotoxicity assay for pure **Orig1**, coated Orig1 and free cHSA. Figure S25: Cadnano map of **Orig1**, **Orig2** and **Orig3**. Table S1: Strands for DNA origamis.
