# Supplementary material for: Cationic Albumin Encapsulated DNA Origami for Enhanced Cellular Transfection and Stability
Source: Materials (Basel). 2019 Mar 21;12(6):949. doi: 10.3390/ma12060949 (PMC6470866; doi:10.3390/ma12060949)
Supplement: Supplementary file 1 [file materials-12-00949-s001.pdf]

materials

MDPI

Supplementary Materials

Instruments

Prime Thermal Cycler for annealing is from Bibby Scientific Ltd. Agarose gel electrophoresis device is from BEIJING LIUYI BIOTECHNOLOGY CO., LTD. Ultrafiltration centrifuge is purchased from Sartorius Centrisart ® G-26C. Auto Gel Imaging Analysis System is from Peiqing Science and Technology Co., Ltd. Concentration of DNA origami was determined by Thermo SCIENTIFIC μDrop™ Plate. Transmission Electron Microscope is from HITACHI HT7700. Atomic Force Microscope is from Bruker MultiMode8 equipped with ScanAsyst mode. Laser Scanning Confocal Microscopy (LSCM) is from Olympus FV 1000.

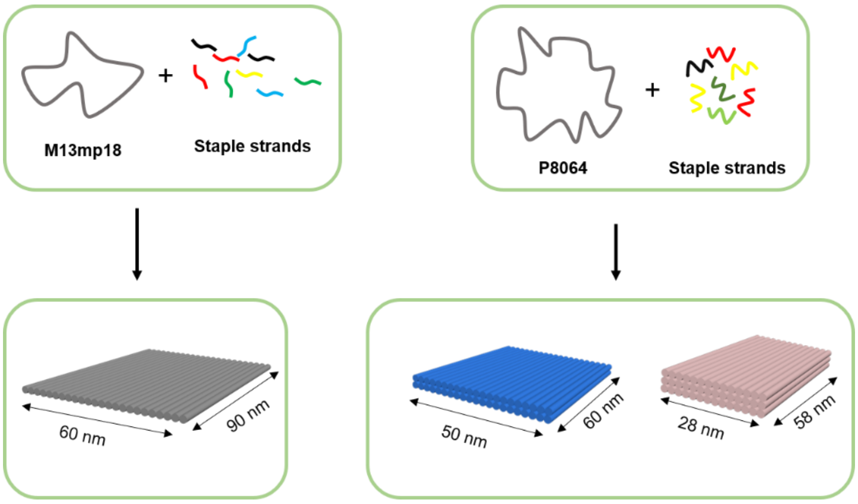

Figure S1. Scheme for DNA origami folding with different structures using different scaffolds.

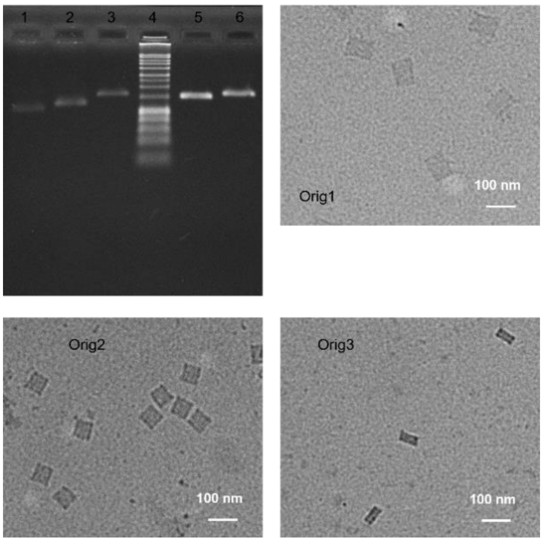

Figure S2. Gel EMSA of purified DNA origami and corresponding TEM imaging. Line 1. **Orig3**, 2. **Orig2**, 3. P8064 scaffold, 4. DNA marker, 5. M13mp18, 6. **Orig1**.

Synthesis procedures of cHSA is referred from previous work [1].

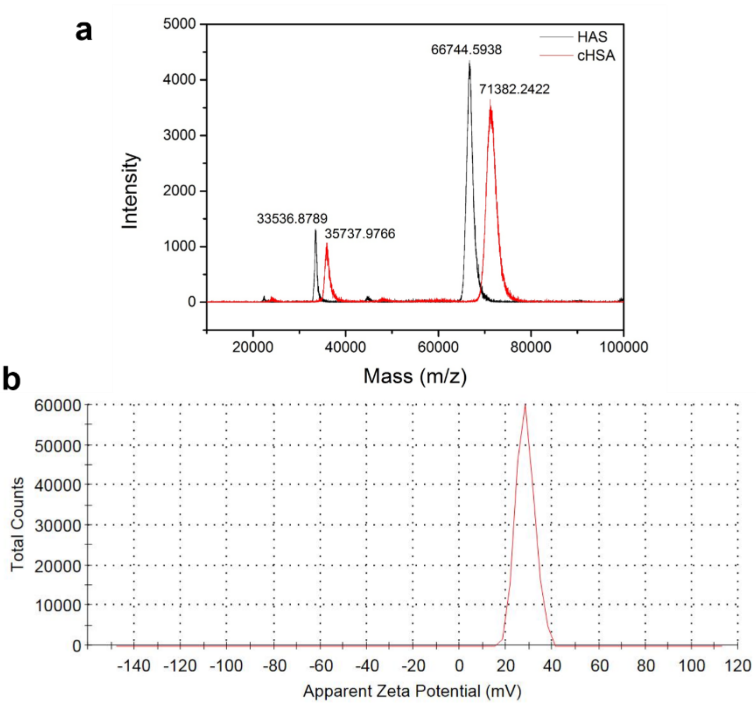

Figure S3. MALDI-Tof spectrum of HSA and cHSA. (a) According to the increasement of the molecular weight, it demonstrated the successful modification of amidogen on HSA. (b) Zeta potential of cHSA.

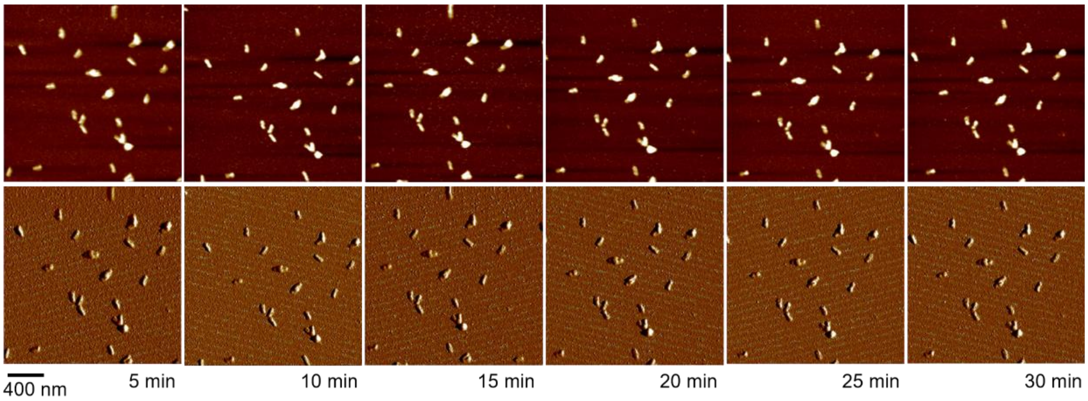

Figure S4. Real time observation of coated **Orig1** using AFM. The coated **Orig1** with ratio 500 was prepared and immediately introduced into the liquid pool, after 5 min deposition, the coated Orig1 was imaged every 5 minutes. The coated origami was observed just in 5 min, and there was no difference when the time was longer.

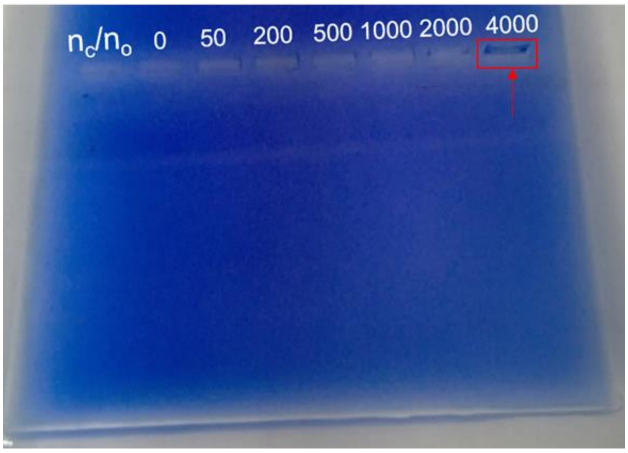

Figure S5. The agarose gel with coated origami was stained with coomassie brilliant blue, then the stained cHSA was observed in the loading well. The coated origami stained with Coomassie Brilliant Blue is shown in red pane, which shows the cHSA protein.

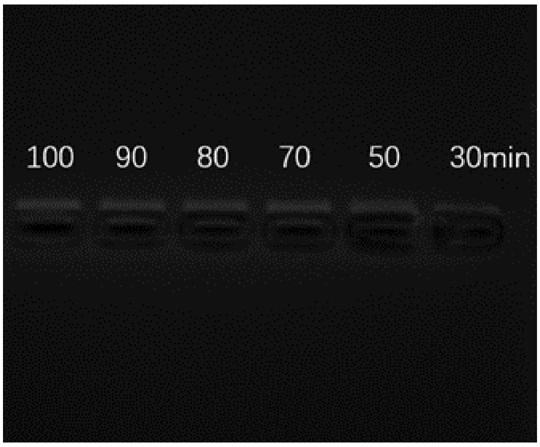

Figure S6. Gel EMSA of pure cHSA. Constant amount of cHSA were respectively loaded in gel well at different running time. The number on the gel represented the final running time of each sample.

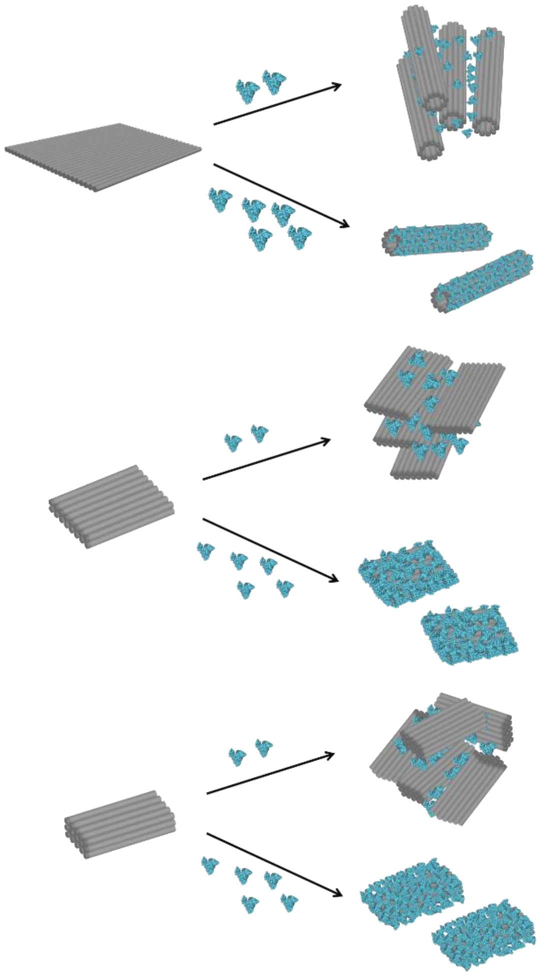

Figure S7. Schematic illustration for coating process of low amount and high amount of cHSA with DNA origami.

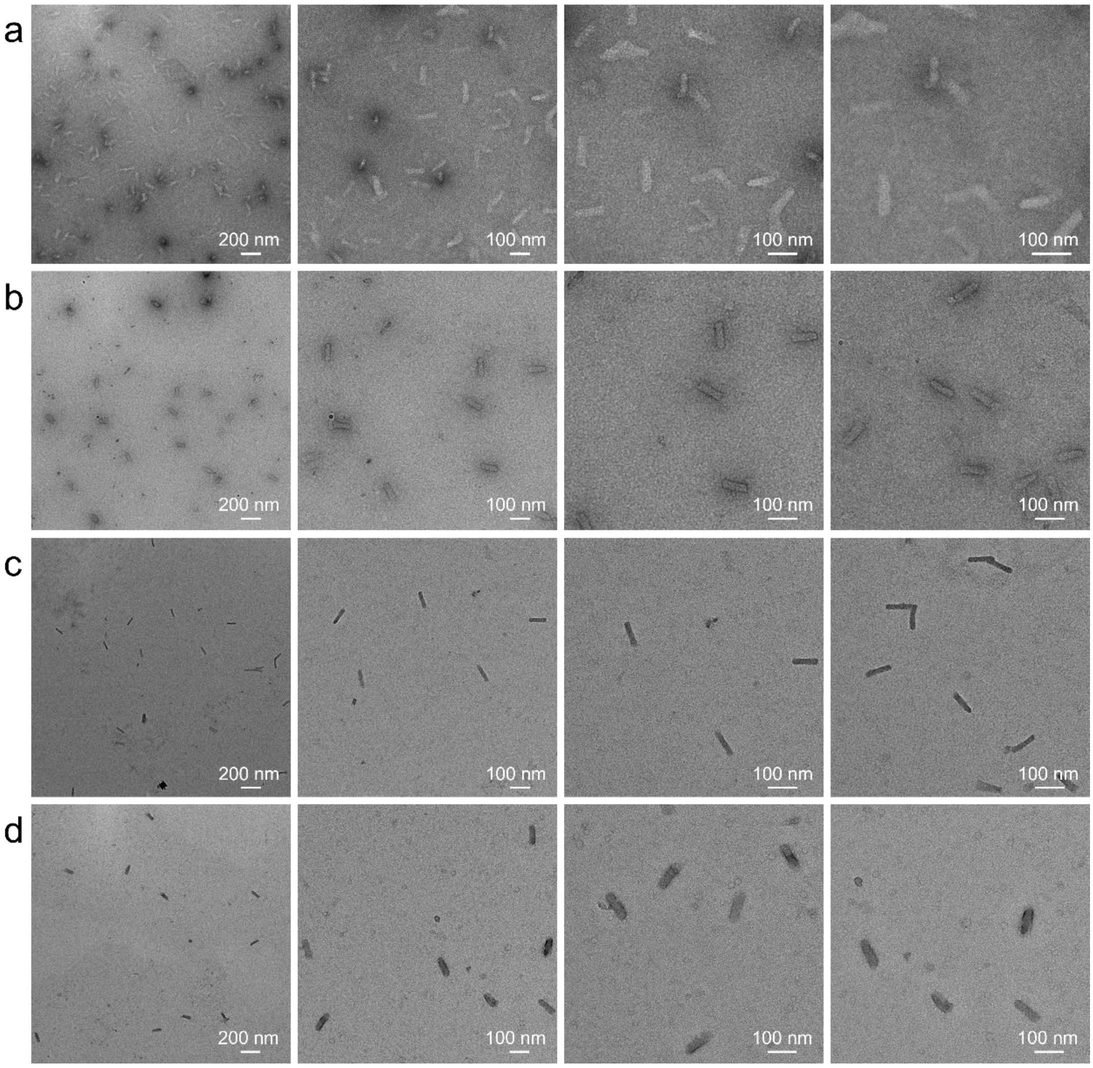

Figure S8. TEM images of **Orig1** coated with increasing amount of cHSA. a, b, c, d: the coated **Orig1** with ratio of 500, 1000, 2000, 4000.

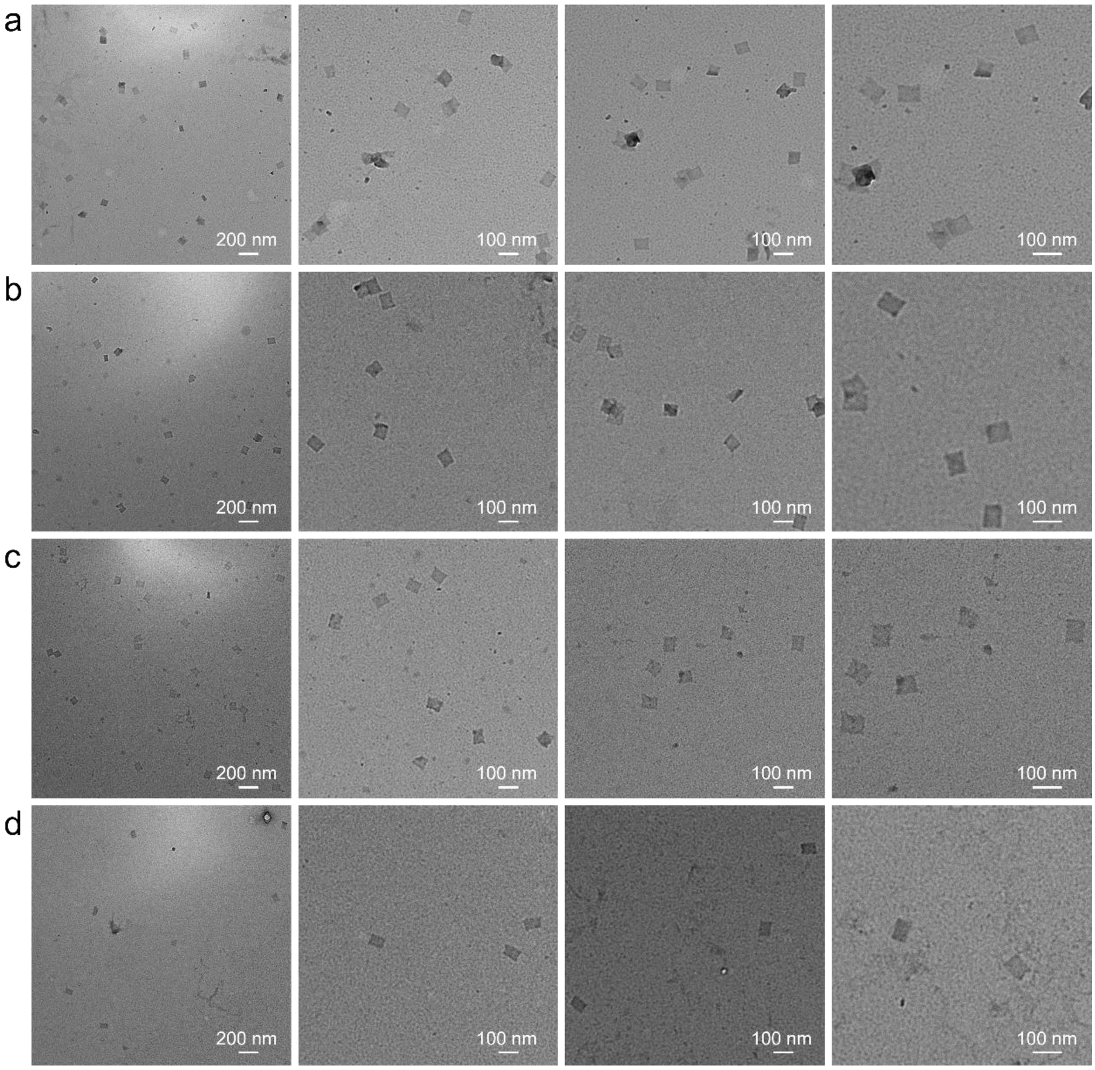

Figure S9. TEM images of **Orig2** coated with increasing amount of cHSA. a, b, c, d: the coated **Orig2** with ratio of 500, 1000, 2000, 4000.

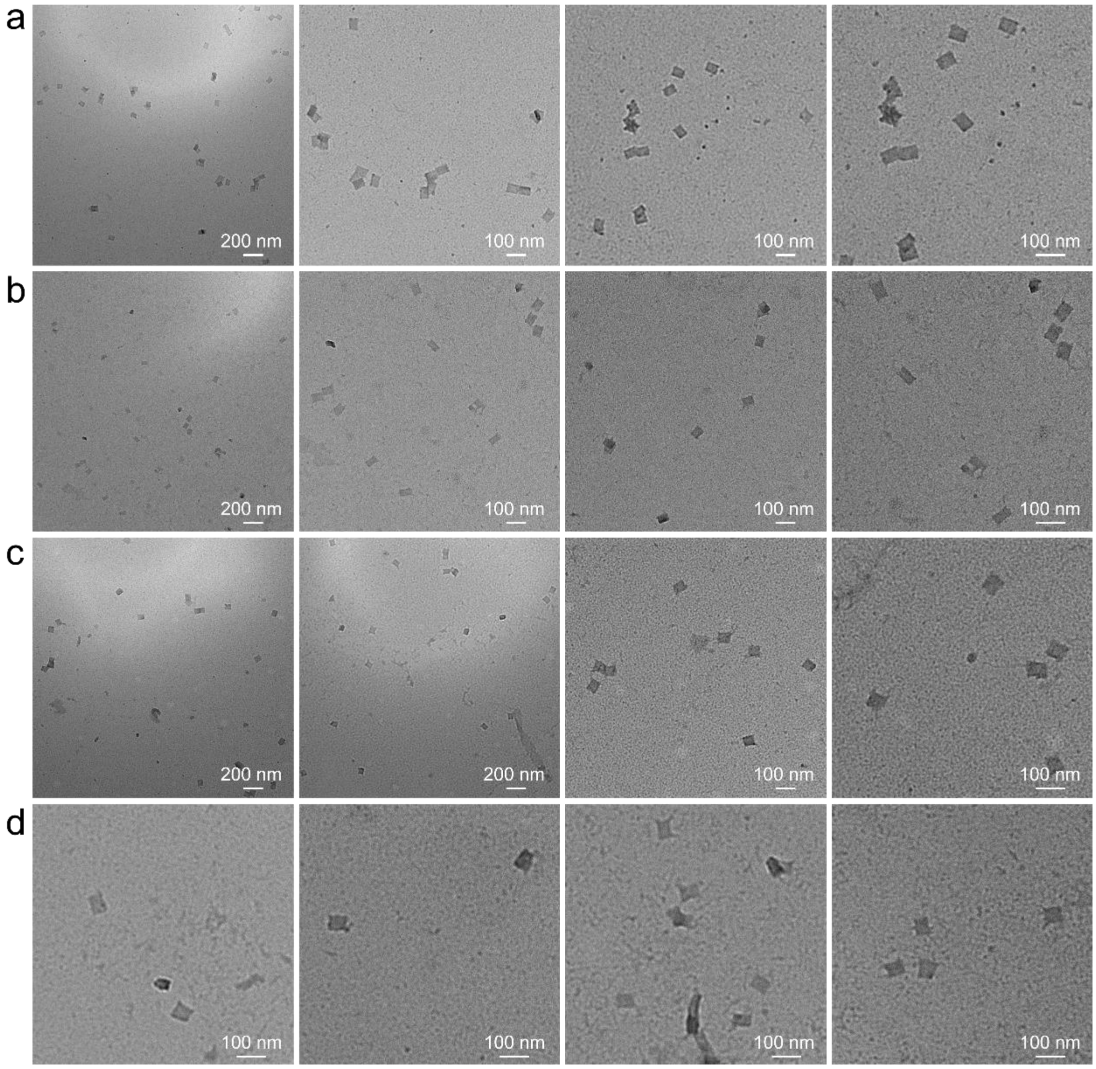

Figure S10. TEM images of **Orig3** coated with increasing amount of cHSA. a, b, c, d: the coated **Orig3** with ratio of 500, 1000, 2000, 4000.

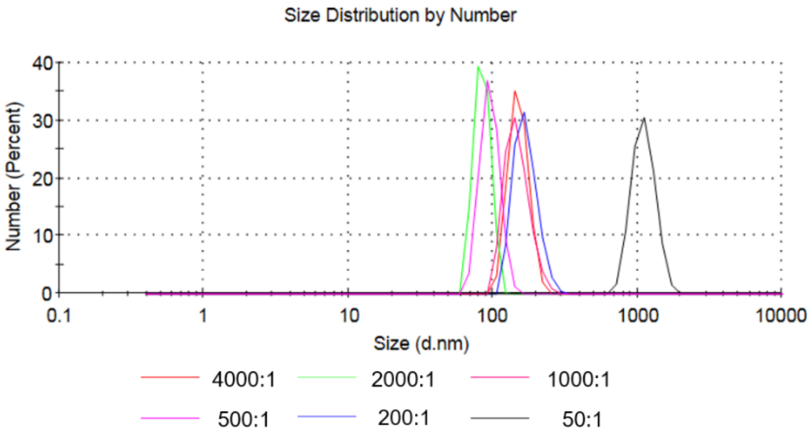

Figure S11. Dynamic light scattering measurement of coated **Orig1** with different cHSA ratios.

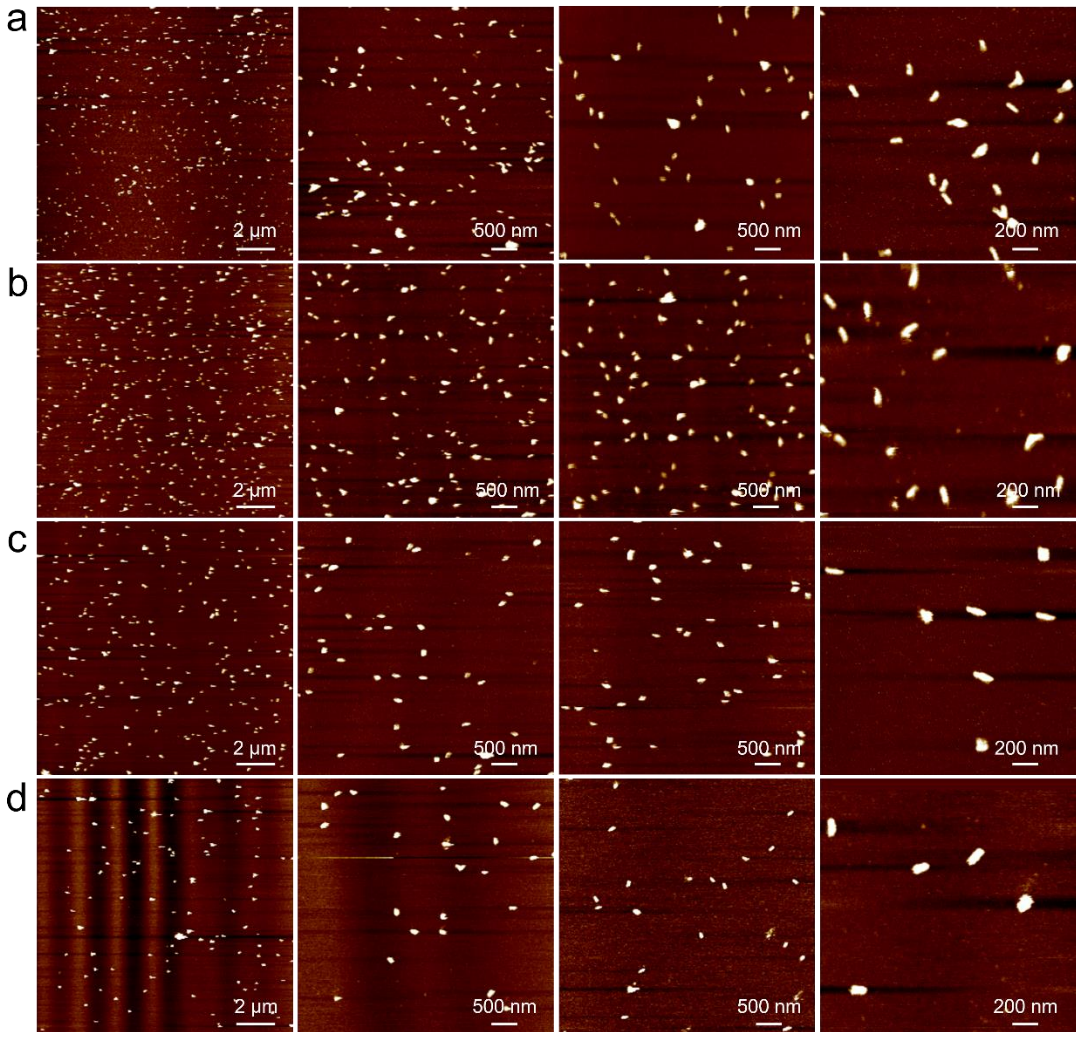

Figure S12. AFM images of **Orig1** coated with increasing amount of cHSA. a, b, c, d: the coated **Orig1** with ratio of 500, 1000, 2000, 4000.

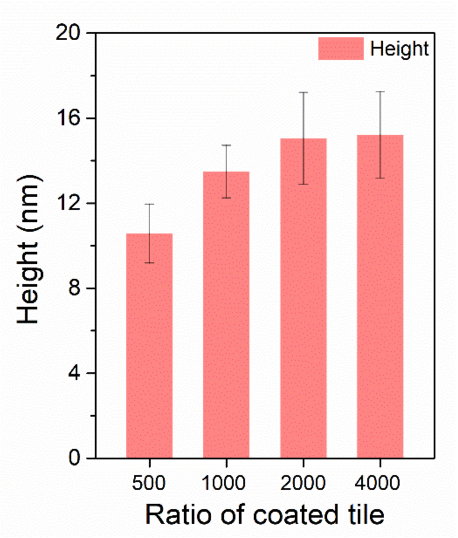

Figure S13. The measured height of coated **Orig1** with ratio of 500, 1000, 2000, 4000. Each histogram data was calculated more than 30 coated origami.

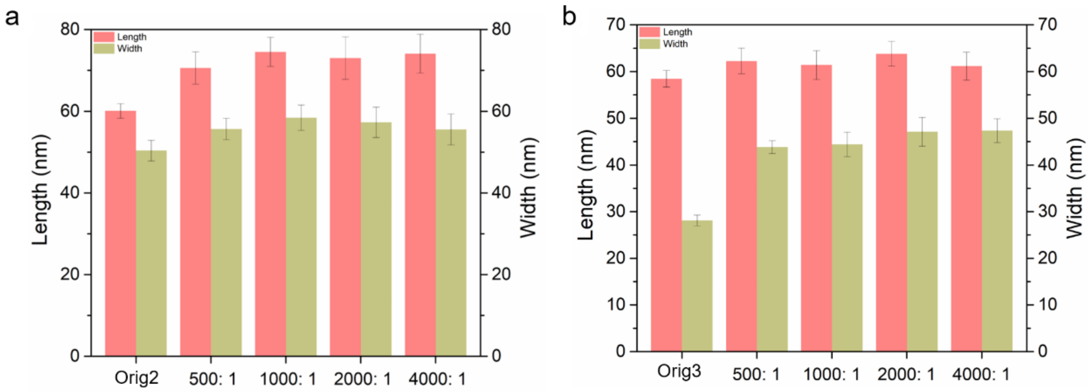

Figure S14. The measured length and width of coated **Orig2** and **Orig3**. a. the length and width of **Orig2** and coated **Orig2**. b. the length and width of **Orig3** and coated **Orig3**. Each histogram data was calculated more than 30 origami and coated origami. The **Orig3** was found mainly increased in width. This is probably due to the DNA origami design. The long edge of **Orig3** is formed by 3 parallel bundles, which could adsorb substantial amount of cHSA. Whereas the short edge is formed by single strand DNA turns, which would absorb less cHSA due to less negative charges. For **Orig2**, the long edge only contains 2 DNA bundles, thus the difference with the short edge is not that significant. The increase both in the length and width was observed (the maximum increase for length is ~20% and for width is ~ 15%).

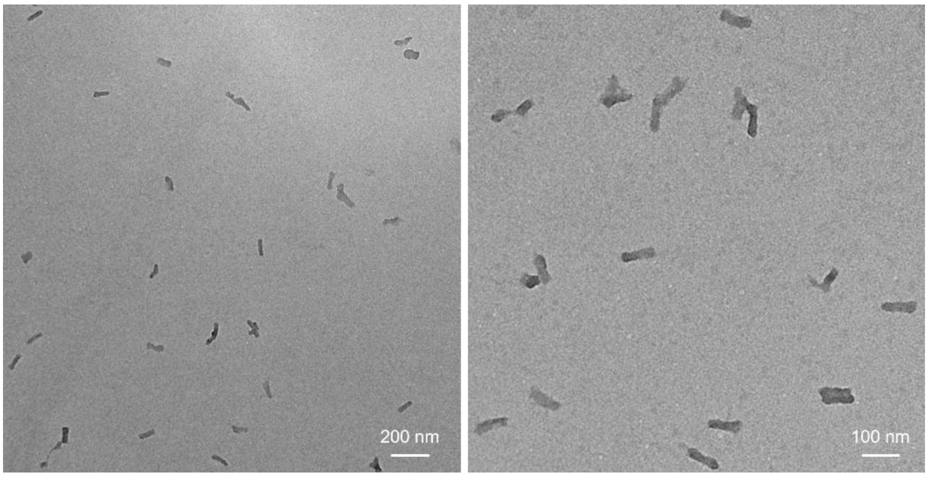

Figure S15. TEM imaging of the coated **Orig1** with ratio of 4000 after two days, the TEM images showed that the coated origami still kept the tube shape.

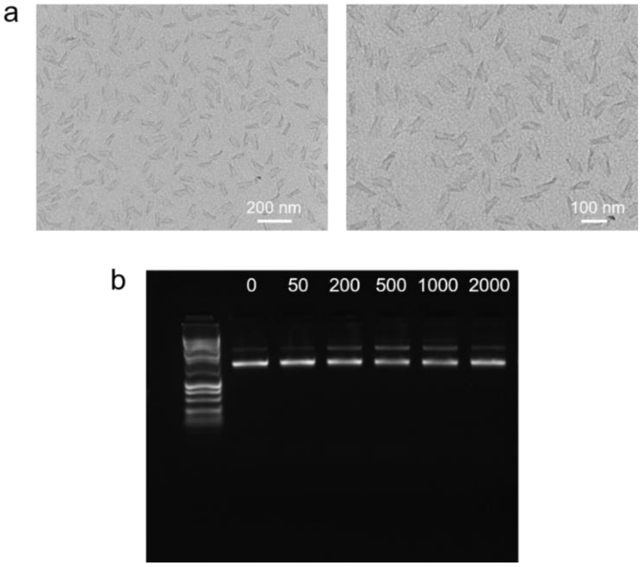

Figure S16. a. TEM images of avidin coated **Orig1** with ratio of 2000, there is no rolling up effect observed; b. Gel EMSA of avidin coated **Orig1** with ratio of 0, 50, 200, 500, 1000, 2000. There is no shift of DNA origami band, it means that there is no coating effect between avidin and origami.

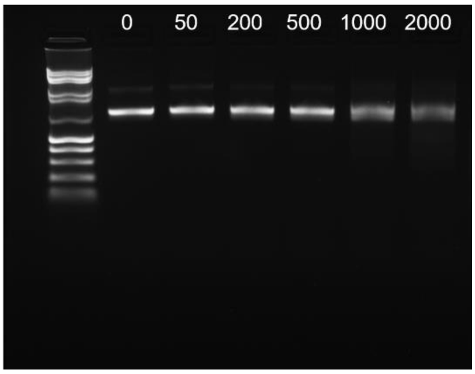

Figure S17. Gel EMSA of native HSA coated **Orig1** with ratio of 0, 50, 200, 500, 1000, 2000. Each sample has plain origami band, and there is almost no shift of origami band.

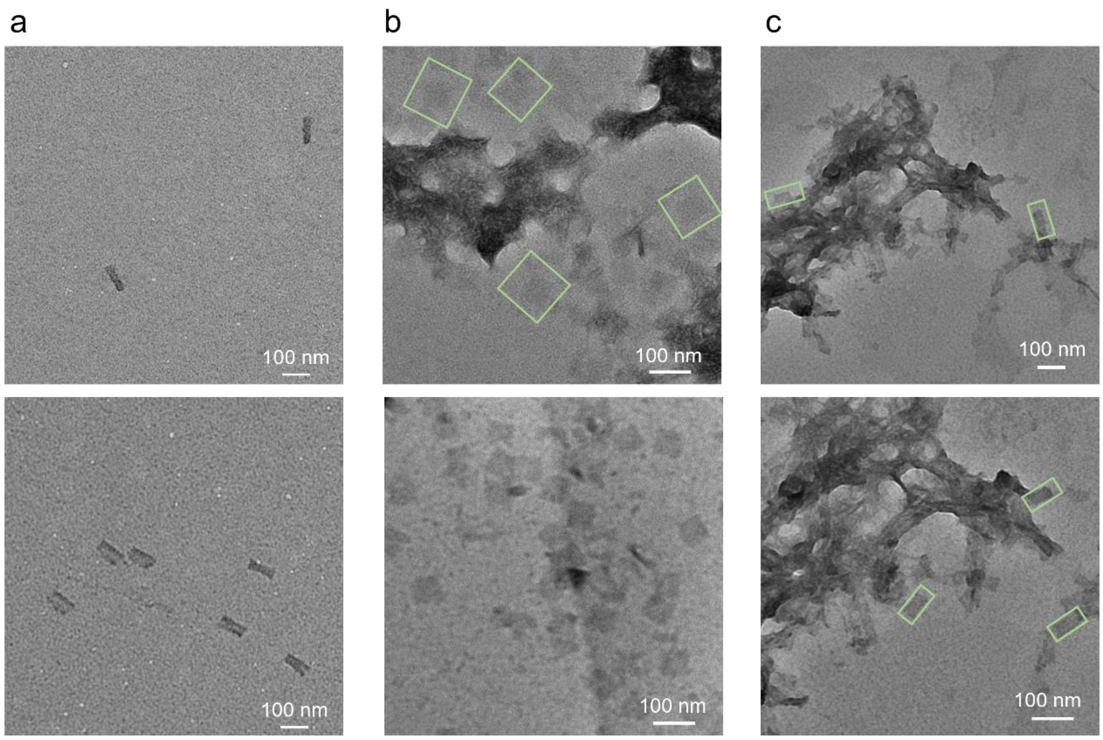

Figure S18. TEM imaging of (a) coated **Orig1**, (b) coated **Orig2** and (c) coated **Orig3** with ratio of 1000 treated with 10 U DNase Ⅰ. The integrated structures were observed in green squares.

The stability of partly coated origami was also verified by using the cHSA coated **Orig1** with nc/no ratio of 50 and 200. Due to the loose interaction and incomplete passivation, the negatively charged DNase I could interact with cHSA and replace them from DNA origami. Therefore, free **Orig1** band was observed with low concentration of DNase I and it can be degraded into small pieces when DNase I increasing to 10 Unit.

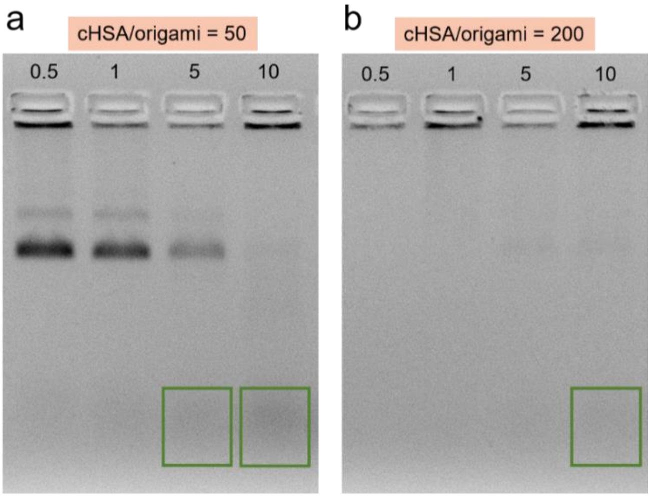

Figure S19. Gel EMSA of coated **Orig1** with ratio of 50 and 200 treated with increasing amount of DNase Ⅰ (0.5-10 U). Small pieces can be observed on the gel when the coating ratio is low.

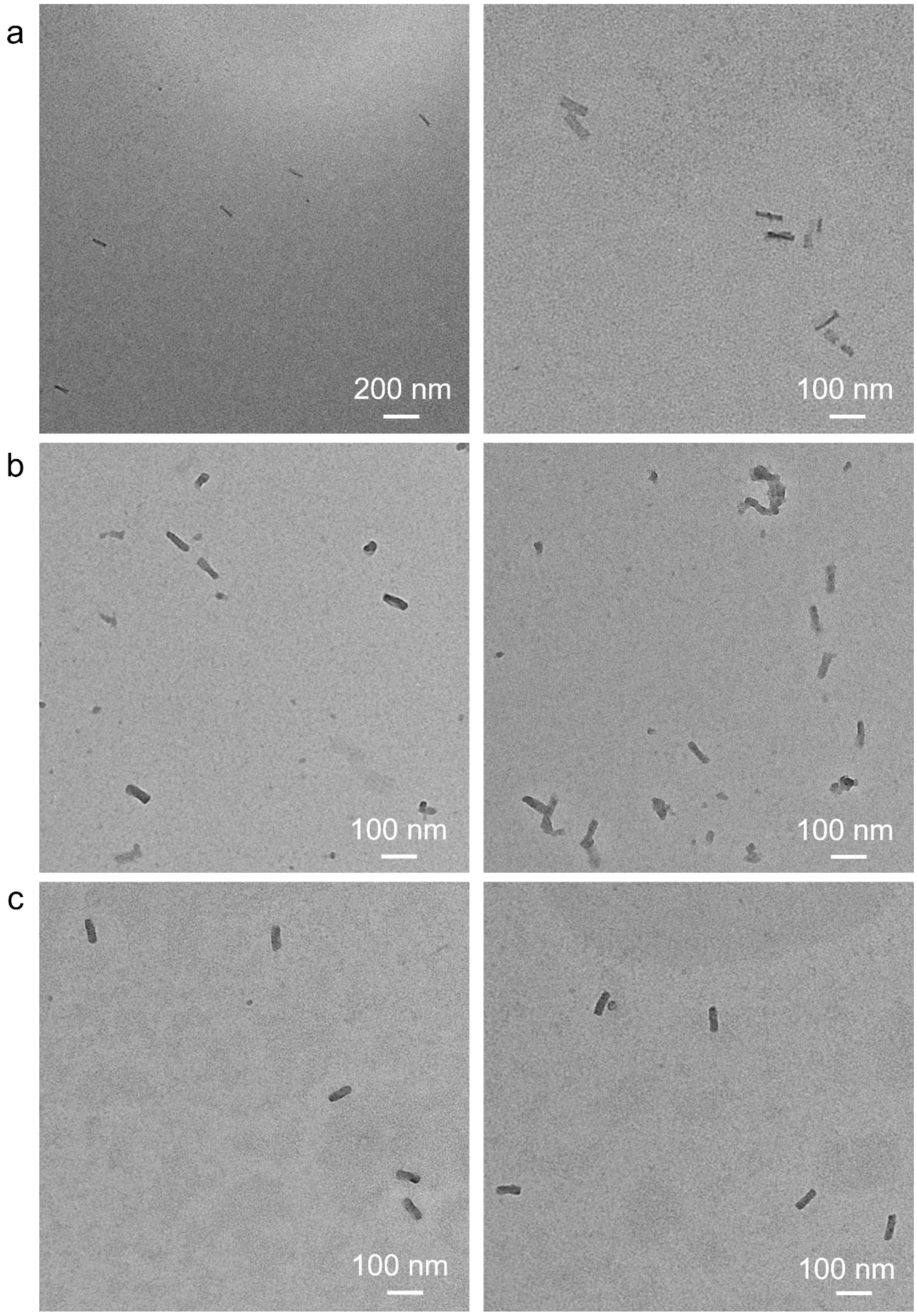

Figure S20. TEM images of coated **Orig1** with ratio 4000 under a. DNase Ⅰ treatement, b. low salt, c. faintly acid conditions.

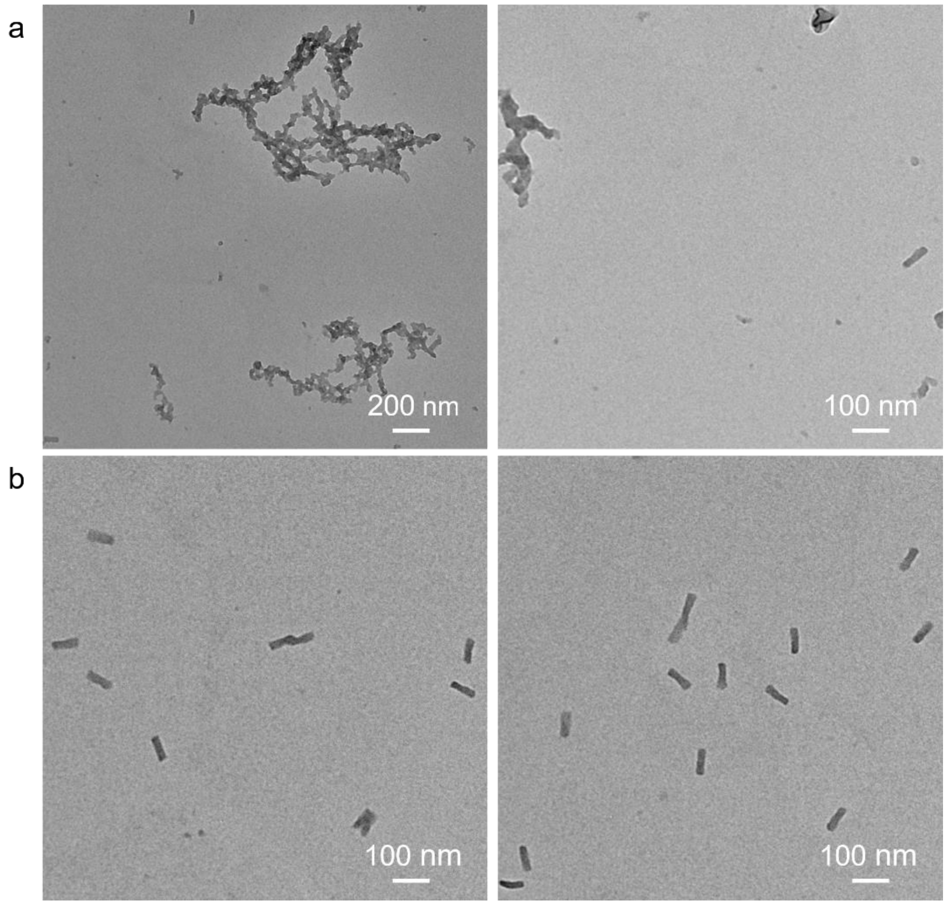

Figure S21. TEM images of coated **Orig1** with ratio 1000 under a. low salt, b. faintly acid conditions treatment.

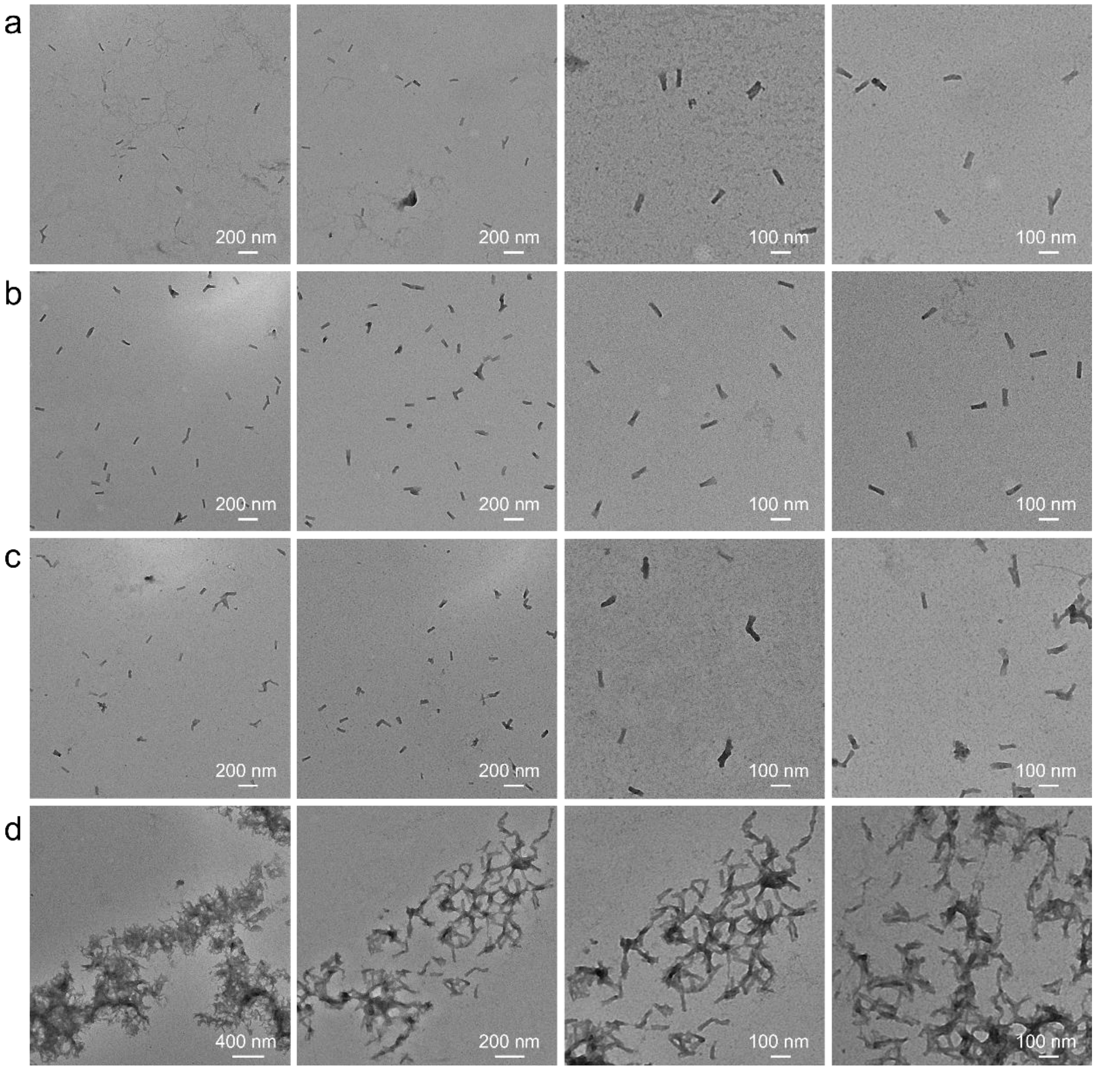

Figure S22. TEM images of coated **Orig1** with different ratios a. 4000, b. 2000, c.1000, d. 500 in cell medium.

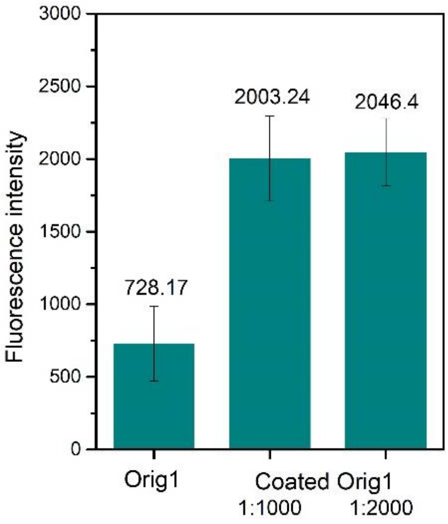

Figure 23. Relative red fluorescence data in cells collected by Image J. The fluorescence of coted origami in cells was obviously increased compared to plain **Orig1**. About 18 cells analyzed by confocal were collected in each sample.

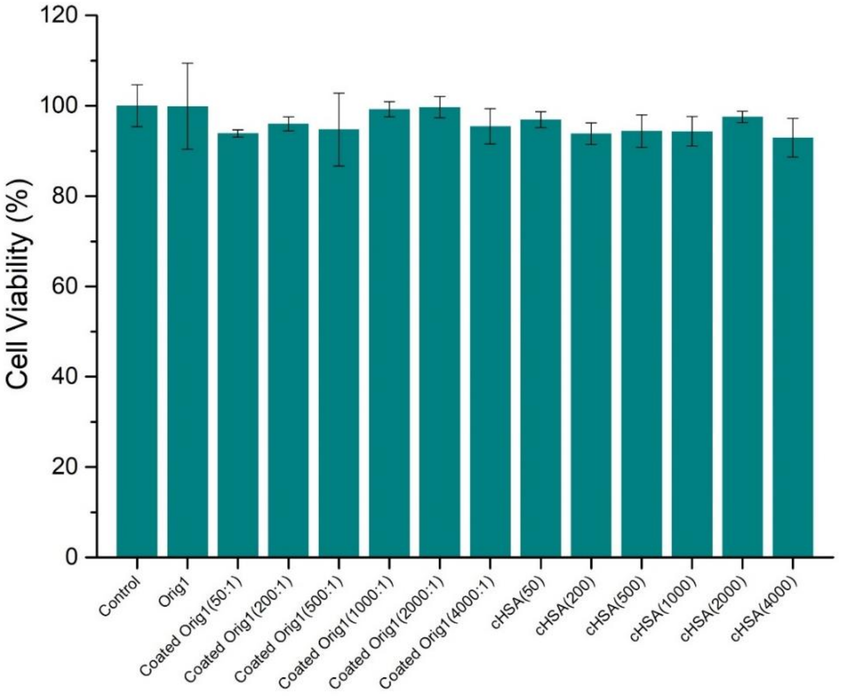

Figure 24. Cytotoxicity assay for pure **Orig1**, coated **Orig1** and free cHSA. 0.1 nM of **Orig1** or coated **Orig1** with different cHSA ratios was tested with Hela cells for 6hrs (the same condition as used for cell uptake experiments). In comparison, free cHSA at the same amount used for coating was also tested (e.g. cHSA(50) means free cHSA with the same amount used for preparing 50:1 ratio of coated **Orig1**). The cell viability was quantified by Cell Titer-Glo™.

Strands for DNA origamis

The complete set of the staple strands for Orig1 are listed in previous work [2]. To avoid the connection between Orig1 DNA origamis, the two lines of staple strands on two short sides were removed. And The complete set of the staple strands for Orig2 and Orig3 are listed in Ref.[3].

11 sticky strands extended from staple strands were complementary to Cy5 labeled strand to introduce Cy5 fluorescent molecule to DNA origami (sequence 5′→3′), sequences are listed in Table S1.

Cy5 labeled strands: 5′-Cy5-CTCTACCACCTAC-3′.

Cadnano map of **Orig1**.

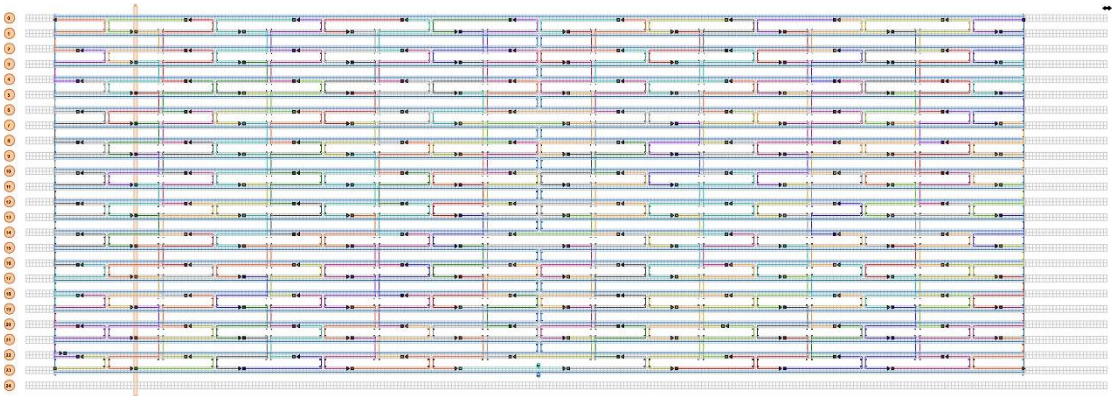

Cadnano map of **Orig2**.

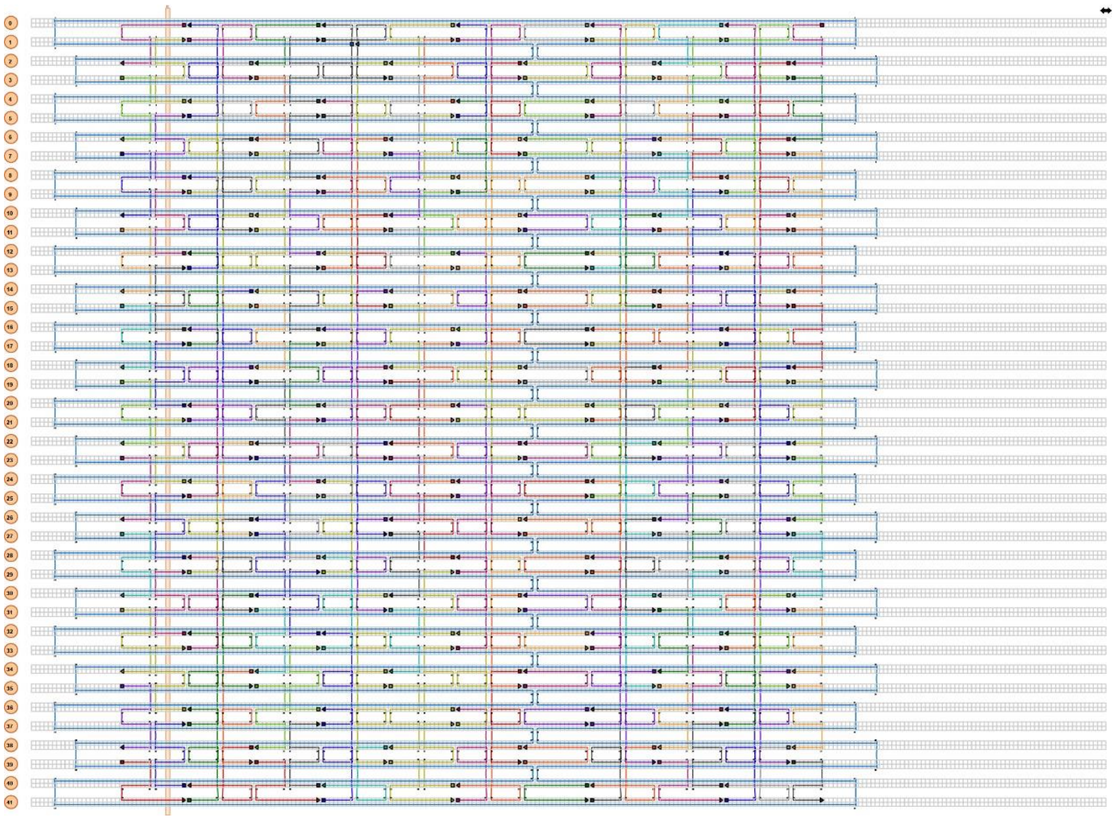

Cadnano map of **Orig3**.

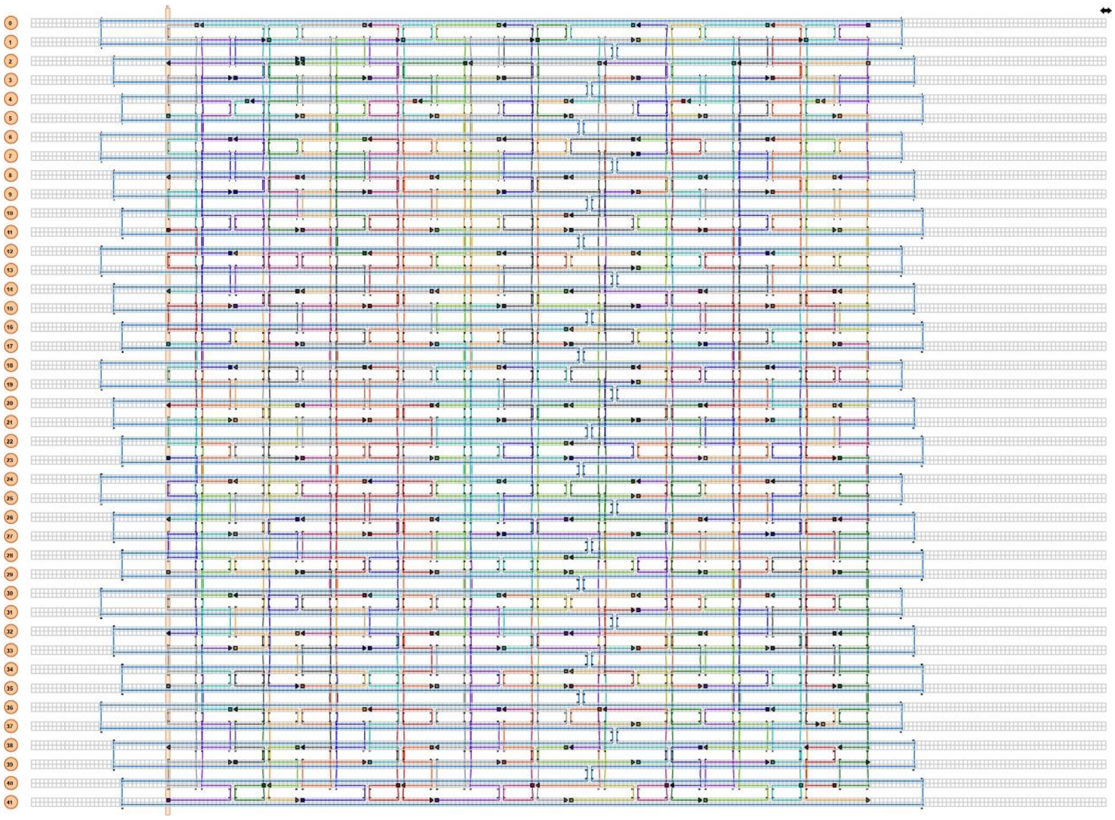

Figure S25. Cadnano map of **Orig1**, **Orig2** and **Orig3**.

Table S1. sticky strands (sequence 5′→3′).

**sticky-11**	CCTAATTTACGCTAAC<br>GAGCGTCTAATCAATATTTTTAGTAGGTGGTAGAG
**sticky-53**	CCTCAAGAATACATGG<br>CTTTTGATAGAACCACTTTTTAGTAGGTGGTAGAG
**sticky-63**	CAAGCAAGACGCGCCT<br>GTTTATCAAGAATCGCTTTTTAGTAGGTGGTAGAG
**sticky-71**	ATTTTGCGTCTTTAGG<br>AGCACTAAGCAACAGTTTTTTAGTAGGTGGTAGAG
**sticky-80**	TGCCTTTAGTCAGACG<br>ATTGGCCTGCCAGAATTTTTTTAGTAGGTGGTAGAG
**sticky-99**	GAAATGGATTATTTAC<br>ATTGGCAGACATTCTGTTTTTTAGTAGGTGGTAGAG
**sticky-158**	AGTTTGGAGCCCTTCA<br>CCGCCTGGTTGCGCTCTTTTTAGTAGGTGGTAGAG
**sticky-166**	GGTAGCTAGGATAAAA<br>ATTTTTAGTTAACATCTTTTTAGTAGGTGGTAGAG
**sticky-176**	CGCCTGATGGAAGTTT<br>CCATTAAACATAACCGTTTTTAGTAGGTGGTAGAG
**sticky-185**	CTTGCATGCATTAATG<br>AATCGGCCCGCCAGGGTTTTTAGTAGGTGGTAGAG
**sticky-204**	ACGTTAGTAAATGAAT<br>TTTCTGTAAGCGGAGTTTTTTTAGTAGGTGGTAGAG

References

1. Wu, Y.; Ihme, S.; Feuring-Buske, M.; Kuan, S.L.; Eisele, K.; Lamla, M.; Wang, Y.; Buske, C.; Weil, T. A Core–Shell Albumin Copolymer Nanotransporter for High Capacity Loading and Two-Step Release of Doxorubicin with Enhanced Anti-Leukemia Activity. *Adv. Healthcare Mater.* **2013**, *2*, 884-894.
2. Chhabra, R.; Sharma, J.; Ke, Y.; Liu, Y.; Rinker, S.; Lindsay, S.; Yan, H. Spatially Addressable Multiprotein Nanoarrays Templated by Aptamer-Tagged DNA Nanoarchitectures. *J. Am. Chem. Soc.* **2007**, *129*, 10304-10305.
3. Ke, Y.; Douglas, S.M.; Liu, M.; Sharma, J.; Cheng, A.; Leung, A.; Liu, Y.; Shih, W.M.; Yan, H. Multilayer DNA Origami Packed on a Square Lattice. *J. Am. Chem. Soc.* **2009**, *131*, 15903-15908.
